# Comparison and Combination of Thermal, Fluorescence, and Hyperspectral Imaging for Monitoring *Fusarium* Head Blight of Wheat on Spikelet Scale

**DOI:** 10.3390/s19102281

**Published:** 2019-05-17

**Authors:** Anne-Katrin Mahlein, Elias Alisaac, Ali Al Masri, Jan Behmann, Heinz-Wilhelm Dehne, Erich-Christian Oerke

**Affiliations:** 1 Institute of Crop Science and Resource Conservation (INRES), Plant Diseases and Plant Protection, Rheinische Friedrich-Wilhelms Universität Bonn, Nussallee 9, 53115 Bonn, Germany; alisaac@uni-bonn.de (E.A.); a.almasri@spatial-business-integration.com (A.A.M.); jbehmann@uni-bonn.de (J.B.); hw-dehne@uni-bonn.de (H.-W.D.); ec-oerke@uni-bonn.de (E.-C.O.); 2 Institute of Sugar Beet Research (IfZ), Holtenser Landstraße 77, 37079 Göttingen, Germany; 3 Spatial Business Integration GmbH, Marienburg 27, 64297 Darmstadt, Germany

**Keywords:** wheat, *Fusarium graminearum*, *Fusarium culmorum*, thermography, chlorophyll fluorescence imaging, hyperspectral imaging, support vector machine, multi-sensor data

## Abstract

Optical sensors have shown high capabilities to improve the detection and monitoring of plant disease development. This study was designed to compare the feasibility of different sensors to characterize *Fusarium* head blight (FHB) caused by *Fusarium graminearum* and *Fusarium culmorum*. Under controlled conditions, time-series measurements were performed with infrared thermography (IRT), chlorophyll fluorescence imaging (CFI), and hyperspectral imaging (HSI) starting 3 days after inoculation (dai). IRT allowed the visualization of temperature differences within the infected spikelets beginning 5 dai. At the same time, a disorder of the photosynthetic activity was confirmed by CFI via maximal fluorescence yields of spikelets (Fm) 5 dai. Pigment-specific simple ratio PSSRa and PSSRb derived from HSI allowed discrimination between *Fusarium*-infected and non-inoculated spikelets 3 dai. This effect on assimilation started earlier and was more pronounced with *F. graminearum*. Except the maximum temperature difference (MTD), all parameters derived from different sensors were significantly correlated with each other and with disease severity (DS). A support vector machine (SVM) classification of parameters derived from IRT, CFI, or HSI allowed the differentiation between non-inoculated and infected spikelets 3 dai with an accuracy of 78, 56 and 78%, respectively. Combining the IRT-HSI or CFI-HSI parameters improved the accuracy to 89% 30 dai.

## 1. Introduction

*Fusarium* head blight (FHB) is one of the most important diseases on small cereals since the beginning of the twentieth century [1]. In recent years, the frequency of FHB has increased because of intensive crop production systems such as intensified maize cultivations [2]. Due to the change in environmental conditions and its impact on pathogen appearance and distribution, improvements in FHB management have a high priority [3]. The fungal pathogens *Fusarium graminearum* Schwabe [teleomorph *Gibberella zeae* (Schwein) Petch] and *Fusarium culmorum* (W.G. Smith) Sacc. are the most dominant pathogens in the FHB disease complex [4,5,6,7]. The high importance of FHB is mainly because of the contamination of infected kernels with mycotoxins, such as trichothecenes (e.g., deoxynivalenol and nivalenol) and zearalenones [8]. During the last decades, research efforts have promoted the resistance level of crop varieties to FHB. However, until now resistance cannot be the only solution to control FHB because of the long time needed to achieve this resistance [9]. Integrated management strategies, in which resistant varieties and fungicides application play a major role, are the best options to control FHB [10]. However, the efficiency of the chemical control of FHB is limited [11]. A critical aspect is scheduling the fungicide treatments during the vegetation period. Periods with a high risk of infection have to be evaluated by epidemiologic models and early infection sites have to be detected with a high accuracy. Suitable techniques are a prerequisite for monitoring crop stands. Besides visible monitoring, optical sensor technologies have been introduced in plant disease monitoring, phenotyping, and precision agriculture [12,13,14,15]. 

Infrared thermography (IRT) is a powerful technique for visualizing, diagnosing, and quantifying plant stresses, resulting from biotic and abiotic stress factors. Thermographic cameras detect the infrared radiation in the range of 7.5–12 µm and display the temperature information in false-color images [14]. The suitability of IRT for early detection of plant diseases has been proved in different studies, e.g., in downy mildew of cucumber and grapevine [16,17,18]. Application of thermal imaging is a promising tool to study spatial patterns of soil-borne pathogens [19,20]. Oerke and Steiner [21] used IRT in the field to detect FHB on winter wheat. They reported a significantly higher temperature of infected spikes compared to the healthy ones. Al Masri et al. [22] used IRT to evaluate FHB development in wheat spikes under greenhouse conditions.

With respect to photosynthetic activity, chlorophyll fluorescence imaging (CFI) was used to detect differences resulting from biotic and abiotic stress. The study of plant diseases such as leaf rust and powdery mildew of wheat and barley have been successfully implemented [23,24,25]. CFI proved a high capability to assess plant stress, including plant diseases, objectively and non-destructively. However, the full capacity of CFI features in field application is difficult because of dark adaptation required prior to measurement, high and varying light intensity [26], and time between emission and detection of the measuring beam [13].

Hyperspectral imaging (HSI) assesses the spectral information as reflectance or transmittance intensity in the visible (VIS, 400–700 nm), near infrared (NIR, 700–1000 nm), and short wave infrared (SWIR, 1000–2500 nm) ranges [14]. The resulting data consists of complex data matrices with three dimensions: two spatial dimensions x and y, and one spectral dimension z. Recently, there has been a remarkable increase in research and reports using hyperspectral imaging in plant disease studies, for example, FHB [15,27], yellow rust [28], powdery mildew of barley [29], *Cercospora* leaf spot, rust and powdery mildew in sugar beet [12,30,31,32], and tomato late blight [33]. These reports concluded that HSI is more objective than the traditional visual methods in characterizing plant diseases, however, these innovative techniques could be further improved [34].

CFI was used in combination with HSI to study FHB and both sensors proved suitable for disease detection after symptoms become visible but this was before senescence [27]. IRT was used in combination with CFI and proved to be very useful for highlighting pre-symptomatic responses of viral diseases and powdery mildew on cucumber [35].

These non-destructive sensors have enabled the accurate and reliable detection of plant diseases and provided new insights into plant-pathogen interactions on different scales [14]. FHB symptoms could be detected by using imaging sensors of thermography, chlorophyll fluorescence, and hyperspectral reflectance after symptoms become visible [2,13,22].

A deeper understanding of FHB using data from multiple sensors is important to optimize risk estimations and management strategies. The aim of the study was to compare IRT, CFI, and HSI for the monitoring of FHB caused by *F. graminearum* and *F. culmorum* under controlled conditions. This individual and multi-sensor data were used as an input for the support vector machine (SVM) classifier to investigate the potential of each combination for early detection of FHB and different stages of pathogenesis. 

## 2. Materials and Methods

### 2.1. Experimental Plants

A variety of spring wheat (*Triticum aestivum* L.) moderately resistant to FHB [36], Passat (KWS, Einbeck, Germany) was used in this study. The spikes of this variety are awnless and light green with 18–22 spikelets in each spike. Pots (12 × 12 × 20 cm) were filled with a mixed substrate (sand, subsoil C, potting substrate ED 73) at a 1:3:6 ratio [v/v/v]). Two seedlings were sown per pot. The plants were supported by sticks and wires to avoid lodging. The plants were cultivated at 20 ± 2 °C and 50–70% relative humidity (RH) in a greenhouse. Artificial light (>300 µmol m^−2^s^−1^, Philips SGR 140, Hamburg, Germany) was used to obtain a photoperiod of 16/08 h day/night.

### 2.2. Inoculum and Inoculation

*Fusarium graminearum*, isolate S.19, and *F. culmorum*, isolate 3.37, were obtained in 2011 and 2004, respectively from infected wheat kernels in Klein-Altendorf, Germany. They were stored at −80 °C in the culture collection of the Institute of Crop Science and Resource Conservation (INRES), University of Bonn. The inoculum was produced on potato dextrose agar (PDA, 39 gL^−1^), potato dextrose broth (PDB, 24 gL^−1^), and low strength potato dextrose agar (LSPDA, 12.5 gL^−1^ and agar-agar 19.5 gL^−1^) according to Moradi [37]. The conidial suspension was adjusted to 10^5^ conidia mL^−1^ using a Fuchs-Rosenthal hemocytometer.

Wheat spikes were inoculated on the same day with each *Fusarium* species separately at GS 61-65 [38]. Four plants with 6 spikes similar in anthesis time were chosen for inoculation. The spikes were inoculated by spraying with 10^5^ conidia mL^−1^ until run-off. Subsequently, plants of each treatment were incubated in separated plastic chambers at optimal conditions for infection (≥95% RH and 22–25 °C),for 48 h. After incubation, plants were grown at 50–70% RH, 16/08 photoperiod, 18/12 °C day/night. Spikes were humidified for 1–2 h per day, using a hand sprayer. Non-inoculated control plants were grown under the same conditions. Six spikelets of each treatment were selected for data analysis.

### 2.3. Disease Severity of FHB

**Figure 1:**
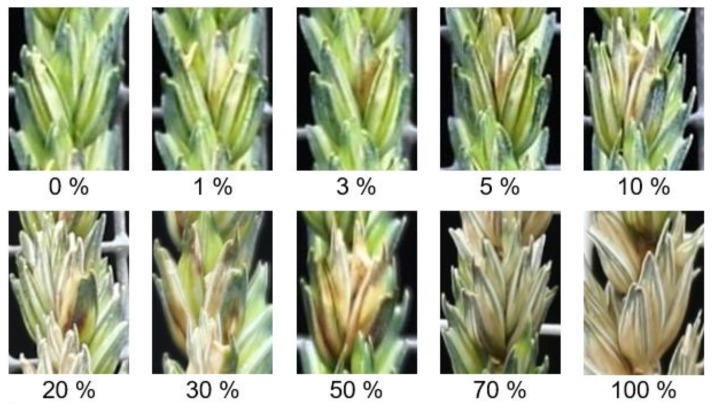
The rating system of disease severity of *Fusarium* head blight (FHB) within a single spikelet of the wheat spike.

In this study, a rating system of FHB severity was established and used for a visual assessment of the disease severity (DS) within each individual spikelet (Figure 1). Disease severity was classified as follows: (i) from 1 to 5% represents early discoloration and small necrotic lesions on glumes, (ii) 10% early bleaching of spikelets which usually cover the typical floret, (iii) 20, 30 and 50% represent combinations of extended necrotic lesions and bleached florets at different levels, (iv) 70% bleached spikelets, but not completely dry, (v) 100% bleached and dry spikelet.

### 2.4. Infrared Thermal Imaging (IRT)

Thermal imaging was performed in a greenhouse under controlled conditions at 50–70% RH and 17–24 °C. The artificial supplementary light was reduced during measuring time to 20 ± 2 µmol m^−2^s^−1^ (Hortilux Schréder, HPS 400W/230V, Monster, Netherland). Spikes were fixed vertically on metal grids (40 × 30 cm, grid size 12 × 12 mm) attached to supporting sticks in the pots. A digital thermocamera (VarioCAM High Definition, Jenoptik, Jena, Germany), sensitive to spectral range from 7.5 to 14 μm with uncooled microbolometer focal plane array was used. The camera has a thermal resolution of 0.03 K at 30 °C, a geometric resolution of 1.024 × 768 IR-pixel, and 30 Hz IR-image frequency. The material emissivity was set to 1 for all measuring dates. The thermocamera was mounted on a tripod and placed at 40 cm distance from spikes and was controlled via the software package IRBIS 3 professional (InfraTec, Dresden, Germany). IR images were analyzed using the same software package by drawing polygons on the selected spikelets, thermal data of ambient air was used for normalization. Maximum temperature difference (MTD) and the average temperature difference between air and spikelets (ΔT) were calculated. All parameters of sensor data are summarized in Table 1.

### 2.5. Chlorophyll Fluorescence Imaging (CFI)

An imaging pulse amplitude modulated chlorophyll fluorometer PAM, MAXI HEAD (Heinz Walz, Effeltrich, Germany) was used for chlorophyll fluorescence measurements under laboratory conditions. The measurement was conducted according to the saturation pulse method immediately after plants had been dark adapted for 15 ± 2 min at room temperature. A standard distance of 18.5 cm between spikes and the camera for a 13 × 15 cm^2^ imagery area was used with a black background. The CCD camera (1392 × 1040 pixel) recorded basic fluorescence F_0_ after illumination of the horizontally laid spikes with blue light (470 nm) of 0.5 µmol quanta m^−2^s^−1^ PAR. Maximum fluorescence (Fm) was recorded after a saturation pulse of 2700 µmol quanta m^−2^s^−1^ PAR for 0.6 s. Based on F_0_ and Fm, the maximal PSII quantum yield (Fv/Fm) was calculated (Table 1) which indicates the capacity of photosynthesis of the spikelets. Saturation pulses of 396 µmol quanta m^−2^s^−1^ PAR were produced at intervals of 20 s until the steady-state condition was reached and the efficient quantum yield Y [II] was estimated which indicates the stability of photosynthesis. The CCD camera was controlled via the software package ImagingWin professional (Heinz Walz, Effeltrich, Germany). Recorded false-color images of Fm, Fv/Fm, and Y [II] were analyzed by drawing polygons that fit the selected spikelets.

### 2.6. Hyperspectral Imaging (HSI)

For hyperspectral imaging, spikes were laid horizontally on a table with a black background (68 cm distance from the cameras) in a light-proof room. Spikes were illuminated with an artificial light source (ASD-Pro-Lamps, Analytical Spectral Devices Inc., Boulder, CO, USA), 50 cm distance from the spikelets, and a vertical inclination of 45°. The hyperspectral camera ImSpector V10E (Spectral Imaging Ltd., Oulu, Finland) was used in the visible-near infrared (VIS-NIR) range from 400 to 1000 nm. A SWIR-camera (ImSpector N25E, Spectral Imaging Ltd., Oulu, Finland) was used in the SWIR from 1000 to 2500 nm. Measurements started after a runtime of 30 min of the entire system. The cameras were focused manually to a white barium sulfate calibration bar with black rhombi (Spectral Imaging Ltd., Oulu, Finland) placed at the same distance of the object. For more details about the imaging setup and image recording see [15].

The software “Processing Imspector 3.1” (Geoscap, Cologne, Germany) was used to calculate the reflectance in relation to a white reference bar and the dark current image. The signals from hyperspectral images were smoothed by applying the Savitzky Golay filter (25 centered supporting points and a third-degree polynomial). All spikelets were masked with HSVaP (“Hyperspectral Visualization and Processing”) [15], and the mean reflectance of each spikelet was extracted with MATLAB R2014a (MathWorks, Natick, MA, USA). Spectral vegetation indices, Normalized Differences Vegetation Index (NDVI), Photochemical Reflection Index (PRI), Pigment-Specific Simple Ratio (PSSRa, PSSRb, PSSRc) and Water Index (WI) (Table 1), were calculated as parameters in order to investigate the potential of specific bands in detecting FHB.

### 2.7. Realization of Measurements

Measurements with the three sensors were performed subsequently on the same day. The thermal imaging was performed under greenhouse conditions from 8:00 to 9:30 am. CFI and HSI measurements took place in a laboratory. Time-series images were performed 3, 5, 7, 12, 17, 21, and 30 days after inoculation (dai). For data analysis, six spikelets were chosen from six spikes as experimental replications for each treatment.

### 2.8. Statistical Analysis

The Superior Performing Software System SPSS 24 (SPSS Inc., Chicago, IL, USA) was used for statistical analysis. A general linear model for repeated measurements was performed on DS (including the disease severity of infected spikelets and the senescence of non-inoculated control), MTD, ΔT, Fv/Fm, Y [II], Fm, NDVI, PRI, PSSRa, PSSRb, PSSRc, and WI. Mean comparisons of treatments were performed using Tukey’s HSD test (significance level *P* ≤ 0.05). A t-test on each single band of the electromagnetic spectrum was performed in Microsoft Excel 2010. A correlation matrix among all indices was applied using RStudio following Pearson’s method (significance level *P* ≤ 0.05). A support vector machine (SVM) classification was run in RStudio to classify non-inoculated and infected spikelets using the parameters derived from each sensor or a combination of different sensor parameters [15]. Seven data sets were investigated: three from each sensor, three from combinations of two sensors (IRT-CFI, IRT-HSI, and CFI-HSI) and one using multi-sensor data (IRT-CFI-HSI). To train the model, half of the data was used as a training data with the radial basis function (RBF) kernel. A five-fold cross-validation was performed on the training data to optimize the parameters cost (C) and gamma (γ). The rest of the data was separated from the training data to evaluate the model.

## 3. Results

### 3.1. Disease Development

**Figure 2:**
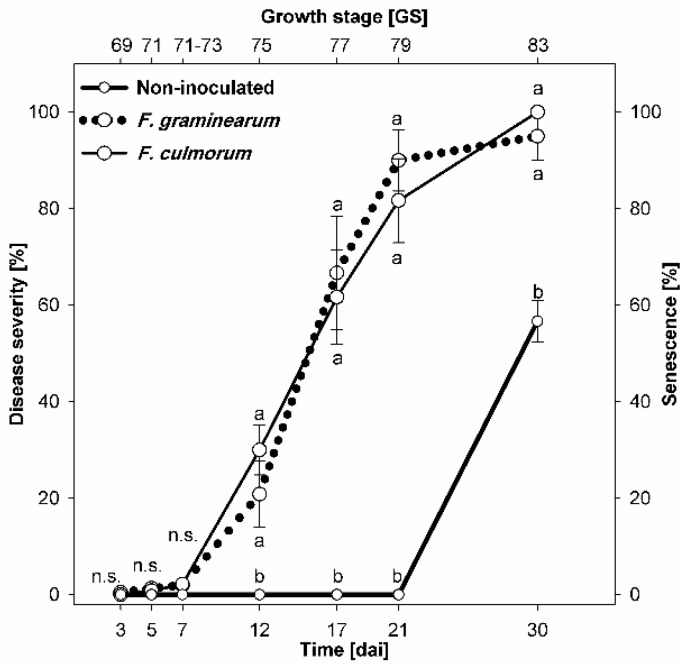
Progress curves of *Fusarium* head blight severity (% diseased spikelet area) due to *Fusarium graminearum* (dotted line) and *Fusarium culmorum* (solid line) on wheat spikelets after spray inoculation compared with non-inoculated control (bold solid line displaying the senescence of non-inoculated control). Spikes were inoculated at GS 61–65. Different letters at the same time point differ significantly according to Tukey’s HSD, P ≤ 0.05 (mean ± SE; n = 6). n.s., not significantly different.

The first symptoms of FHB became visible 3 dai with small necrotic lesions (1%) on glumes of spikelets infected with *F. graminearum*. Starting 5 dai, *F. culmorum* infected spikelets were associated with visible symptoms, not significantly different from those infected with *F. graminearum*. FHB symptoms of both pathogens increased significantly on 7 dai with no statistical difference between the two *Fusarium* species (Figure 2). Natural senescence started on 21 dai on non-inoculated spikelets, and could be monitored easily by visual assessment, the non-inoculated spikelets showed an average bleaching of 56% 30 dai (GS 83) (Figure 2).

### 3.2. Effect of Fusarium Infection on Spikelet Temperature

**Figure 3:**
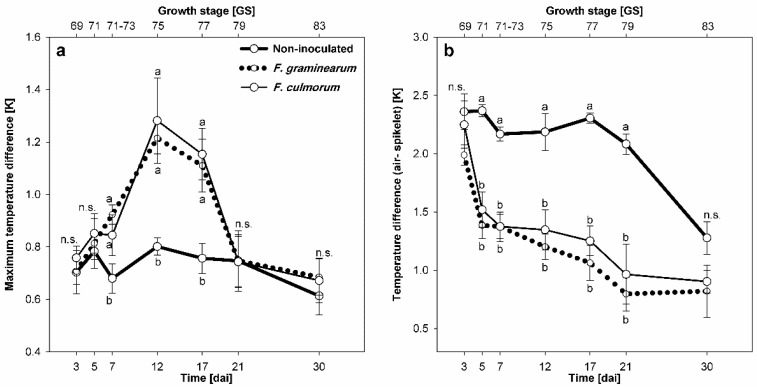
Effect of FHB caused by *F. graminearum* (dotted lines) and *F. culmorum* (solid lines) on the development of maximum temperature difference (**a**) and average temperature difference [ΔT = air – spikelet] (**b**) compared with non-inoculated control (bold solid lines). Spikes were spray-inoculated at GS 61–65. Different letters at the same date differ significantly according to Tukey’s HSD, P ≤ 0.05 (mean ± SE; n = 6). n.s., not significantly different.

*Fusarium* species affected the temperature of infected spikelets compared with non-inoculated control ones. The MTD (Figure 3a) indicates the temperature heterogeneity within individual spikelet. Significant increases in MTDs were observed starting 7 dai for infected spikelets of both *Fusarium* species compared with non-inoculated control (Figure 3a). Maximum MTDs were observed 12 dai, then the infected spikelets showed lower temperature heterogeneity. At 21 dai, MTDs of infected spikelets were not significantly different from the non-inoculated control (Figure 3a). A reduced ΔT of *Fusarium*-infected spikelets compared with non-inoculated control was observed 5 dai and was significant until 21 dai. In general, ΔTs of non-inoculated control were higher than those of infected spikelets with each pathogen even up until 30 dai.

### 3.3. Effect of Fusarium Infection on Chlorophyll Fluorescence

*Fusarium* infection caused by both *Fusarium* species was associated with reducing the photosynthetic activity of spikelet tissue in early stage of infection. In advanced stages (i.e., bleached symptoms stage), the photosynthetic apparatus was completely destroyed.

**Figure 4:**
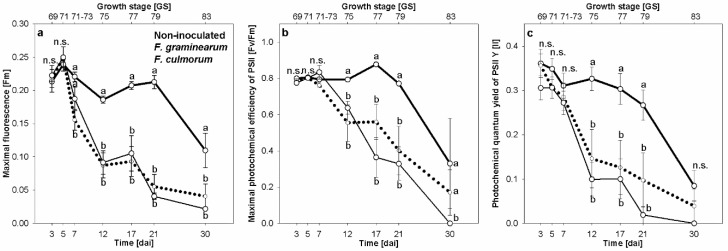
Effect of *Fusarium* head blight caused by *F. graminearum* (dotted lines) and *F. culmorum* (solid lines) on maximal fluorescence of dark-adapted spikelets [Fm] (**a**), maximal photochemical efficacy of photosynthesis II [Fv/Fm] (**b**), and photochemical quantum yield Y [II] (**c**) in comparison to non-inoculated control (bold solid lines). Spikes were spray inoculated at GS 61–65. Tukey’s HSD, P ≤ 0.05 (mean ± SE; n = 6). n.s., not significantly different.

The Fm of dark-adapted spikelets was significantly reduced in *F. graminearum* infected spikelets compared with *F. culmorum* infected and non-inoculated control spikelets 7 dai (Figure 4a). The infection with each *Fusarium* species reduced Fm significantly after 7 dai compared with non-inoculated control. These differences were also pronounced 30 dai. Neither maximal photochemical efficacy of photosynthesis II [Fv/Fm] nor photochemical quantum yield Y [II] were suitable parameters to differentiate between infected spikelets and non-inoculated ones during the first week after inoculation (Figure 4b,c). Fv/Fm and Y [II] were significantly reduced only 12 dai as compared with non-inoculated control. At the last time point of the assessment, 30 dai, Fv/Fm of *F. culmorum* infected spikelets was significantly different from *F. graminearum* infected spikelets and those of the non-inoculated control (Figure 4b). In contrast, the Y [II] showed no significant differences among all treatments 30 dai (Figure 4c).

### 3.4. Effect of Fusarium Infection on Spectral Signature of Spikelets

**Figure 5:**
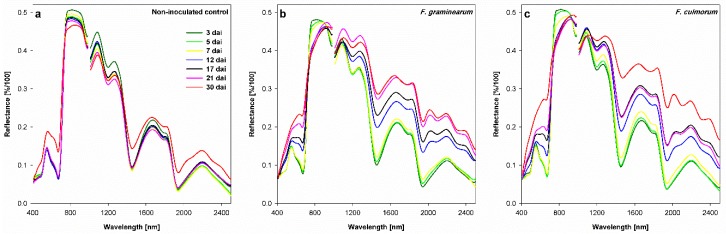
Effect of *Fusarium* head blight caused by *F. graminearum* (**b**) and *F. culmorum* (**c**) on spectral signatures of wheat spikelets compared with the non-inoculated control (**a**) at different time points after inoculation (3–30 days past inoculation, dai). Spikes were spray inoculated at GS 61–65. Means; n = 6.

The spectral signatures of the non-inoculated control showed minor changes during the first 6 measuring times (until 21 dai). The senescence of the non-inoculated control increased the reflectance in the VIS and the SWIR ranges 30 dai (Figure 5a). A lower reflectance was observed in the NIR (Figure 5a). The spectral signatures of FHB infected spikelets changed considerably in comparison with the non-inoculated control parallel to the development of infection (Figure 5). The reflectance of FHB spikelets showed gradual but low changes along the entire spectral signature until 7 dai for both *Fusarium* species (Figure 5b,c). More pronounced changes were shown for *F. culmorum* infected spikelets in the SWIR range. From 12 dai onwards, the shape of spectra showed distinct changes compared with the earlier measurement times. Here, higher reflectance in the VIS and SWIR ranges and lower reflectance in the NIR range was pronounced. The largest changes in the shape of the spectral signatures were detected in the VIS range starting 17 dai. In the NIR range, the decrease in the spectral reflectance started 12 dai. At 30 dai, the strongest alteration was measured especially for spikelets infected with *F. culmorum* (Figure 5b,c).

**Figure 6:**
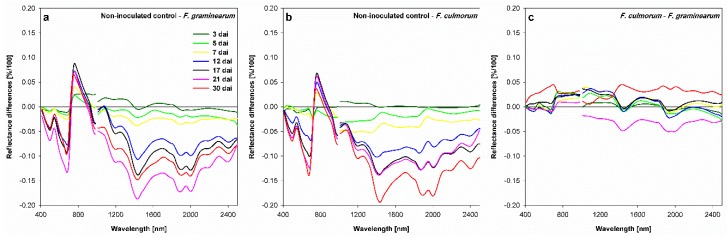
Difference spectra of *Fusarium* head blight caused by *F. graminearum* to non-inoculated control (**a**), FHB caused by *F. culmorum* to non-inoculated-control (**b**), and FHB caused by both *Fusarium* species separately (**c**) at different time points of assessment after inoculation. Spikes were spray inoculated at GS 61-65. Means; n = 6.

Differences between non-inoculated control and *Fusarium*-infected spikelets were more obvious by plotting the differences of spectral reflectance (Figure 6). The comparison between the non-inoculated control and *F. graminearum* infected spikelets (non-inoculated control—*F. graminearum* infected spikelets) (Figure 6a) showed that the alteration in spectral signature started already at 3 dai. These differences were pronounced around 500 and 675 nm in the VIS range with two negative peaks, and at 760 nm in the NIR range with a positive peak. Wavelengths indicating water content in the SWIR showed clear differences with three negative peaks at 1440, 1880, and 2000 nm. Over time, the reflectance difference increased very clearly. *F. culmorum* infected spikelets showed similar patterns of reflectance difference curves compared with that of *F. graminearum* (non-inoculated control*—F. culmorum* infected spikelets) (Figure 6b). This indicates that the same wavelengths were affected by both *Fusarium* species. The differences between *Fusarium* species in terms of the spectral reflectance (*F. culmorum*–*F. graminearum*) did not exceed ± 0.05% (Figure 6c). It showed that differentiation among causing pathogens of FHB was possible starting at 3 dai. The differences were shown along the spectrum over time. The significance of these differences among the spectral signatures of the treatments was confirmed by a t-test. The differences between spikelets of the non-inoculated control and *F. graminearum* or *F. culmorum* infected spikelets were significant in the VIS and SWIR ranges starting from 7 and 5 dai, respectively. No significant differences could be detected between the spectral signatures of *F. graminearum* and *F. culmorum* infected spikelets.

### 3.5. Correlation between Parameters Derived from Different Sensors

**Figure 7:**
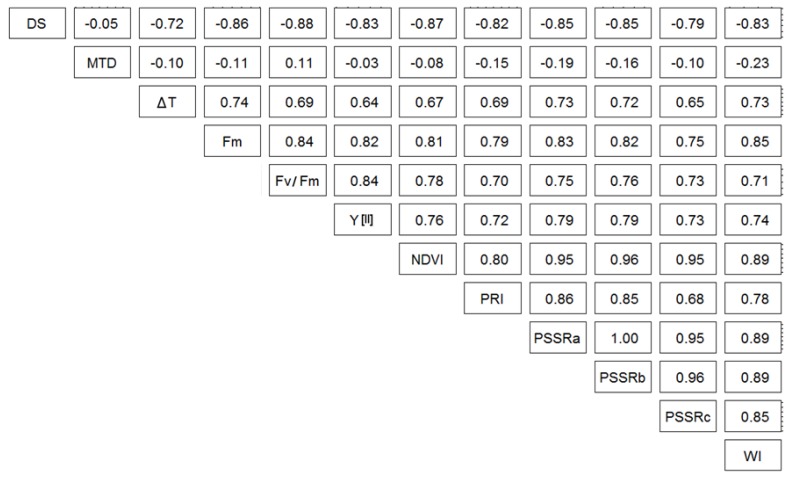
Correlation between disease severity (DS: diseased spikelet area %) and data from different sensors: temperature difference between air and spikelet (ΔT), maximum temperature difference (MTD), maximal fluorescence yields of dark-adapted spikelets (Fm), photochemical quantum yield at steady state Y[II], maximal photochemical efficacy of photosynthesis II (Fv/Fm), normalized difference vegetative index (NDVI), photochemical reflectance index (PRI), pigment specific simple ratio (PSSRa, b, and c) and water index (WI). n = 126 pairs, Pearson method.

Parameters derived from different sensors showed a high significant correlation to each other and to the disease severity according to the Pearson method (Figure 7). MTD was the only parameter that showed no significant correlation to the others. The correlation varied between strong (r from 0.60 to 0.79) and very strong (r ≥ 0.80). The Pearson correlation method confirmed a positive correlation among all selected parameters except MTD, and a negative correlation between DS and sensors data (the strongest, −0.88 to Fv/Fm and the weakest, −0.72 to ΔT).

### 3.6. Spatio-Temporal Dynamics of Fusarium Head Blight

**Figure 8:**
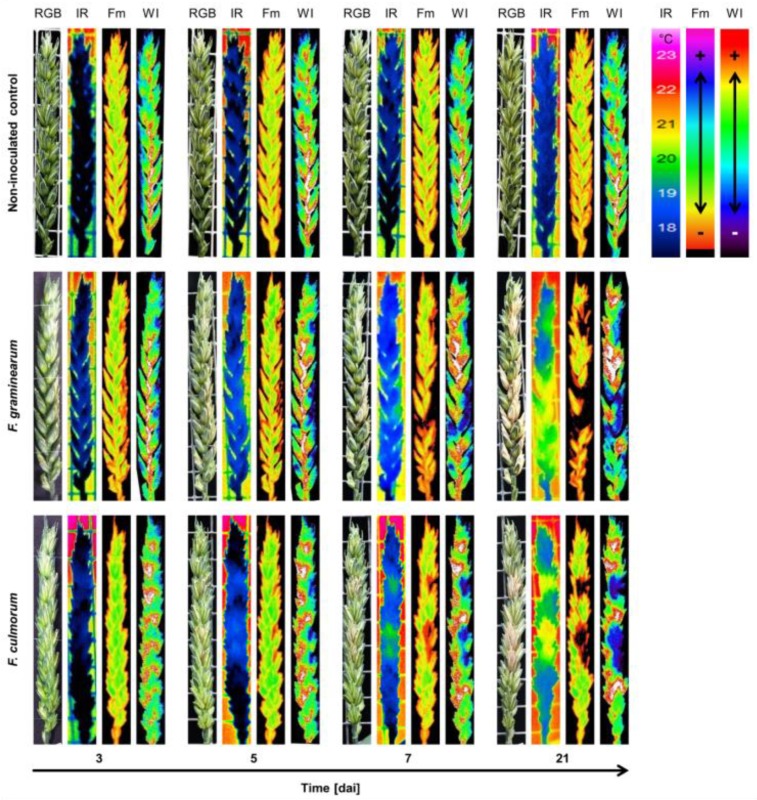
Spatio-temporal dynamics of *Fusarium* head blight caused by *F. graminearum* and *F. culmorum* compared with non-inoculated control visualized by digital images (RGB), thermograms (IR), maximum chlorophyll fluorescence false-color image (Fm), and water index false-color image (WI) derived from spectral reflectance.

Early and late symptoms of FHB compared with the non-inoculated control spikes are shown in Figure 8. The infection development can be visibly distinguished 3 dai with the help of the non-invasive sensors. The temperature of infected spikelets increased and reached values close to those of the ambient air 5 dai for the infection with both *Fusarium* species. At a late infection stage, 21 dai, infected spikelets were completely bleached and showed the temperature with minimum differences to ambient air temperature. The chlorophyll fluorescence index Fm indicated to the spot where the infection started 3 dai, especially on *F. graminearum* infected spikelets. Over time, Fm dropped to zero 21 dai. Early symptoms of FHB were detectable by the WI derived from hyperspectral images starting 3 dai (Figure 8).

Vegetation indices calculated from the spectral reflectance of different treatments are shown in Table 2. NDVI of non-inoculated control was in the range from 0.55–0.77 during the measuring period. Starting at 12 dai, the NDVI was significantly different from the infected spikelets. In contrast, the PRI was significantly different from infected spikelets at an earlier stage, 7 dai, but showed no significant difference to the non-inoculated control at late infection stages. PSSRa and PSSRb differed between non-inoculated control and *F. culmorum* infected spikelets 3 dai. At 5 dai, these indices were significantly different between *Fusarium* infected spikelets. PSSRa, PSSRb and PSSRc, showed significant differences between non-inoculated control and *Fusarium* species infected spikelets 30 dai. WI was similar to PRI in differentiating between non-inoculated control and *Fusarium* infected spikelets, however, WI could differentiate between them even at 30 dai (Table 2).

### 3.7. Support Vector Machine Classification of Infected and Non-Infected Spikelets at Different Pathogenesis Stages

Sensor data (i.e., MTD and ΔT from IRT; Fm, Fv/Fm, and Y [II] from CFI; and NDVI, PRI, PSSRa, PSSRb, PSSRc, and WI from HSI) were used as input parameters in a two-class classification (non-inoculated/infected spikelets) using a SVM approach (Table 3). An accuracy of 78% was obtained 3 dai using the parameters derived from IRT or HSI. This accuracy varied between 78 and 100% depending on the disease stage and decreased to 67% 30 dai using the IRT parameters. The accuracy increased to 100% 12, 17, and 21 dai and decreased to 78% on 30 dai using HSI parameters. Lower accuracy has been obtained when using the CFI parameters as an input of SVM. An accuracy of 56% was observed 3 dai and the maximum accuracy was 89% 7, 12, 17, and 21 dai, then it decreased to 78% 30 dai (Table 3).

Fusing parameters from two sensors showed no improvement in the classifier accuracy in all combinations along the time of the experiment until 21 dai. However, fusing IRT or CFI with HSI parameters increased the accuracy to 89% 30 dai. The best performance of the combinations was achieved using the combination of IRT with HSI with a mean accuracy of 90% over the time of the experiment. Multi-sensor data (i.e, IRT, CFI, and HSI) did not improve the accuracy of the classifier, and the mean accuracy of multi-sensor data was 87% over the time of the experiment (Table 3).

## 4. Discussion

Optical sensors and non-invasive methods have recently attracted an increasing interest [45,46]. They are expected to play a major role in detecting and monitoring plant diseases in the coming years [47]. An application of these technologies to monitor FHB might contribute significantly to secure cereal production systems. Early detection and objective monitoring of FHB using proximal sensors such as IRT, CFI, and HSI, individually or in combination enhance our knowledge to improve the disease management.

FHB symptoms are normally associated with a relatively low water content of infected spikelets. This reduction in water content causes an increased temperature of the infected spikelets compared with the non-inoculated control. This can be attributed to reduced transpiration due to reduced water supply [22]. IRT was successfully used in detecting and monitoring FHB under field and controlled conditions [13,22]. In the current study, *Fusarium*-infected spikelets showed higher temperatures compared with non-inoculated control. This is in accordance with [13,22], where thermal images were analyzed considering the entire spikes. Impeding the movement of assimilates above the preliminary site of infection leads to a reduction in transpiration in the upper part of the spike. Additionally, the “plug of” of the rachilla when the *Fusarium* infection moves from floret to rachilla can have a similar effect [48]. This reduction leads to a higher temperature in that part of the spike [22]. The higher temperature of infected spikelets allowed detection of FHB at the early stages at 5 and 7 dai based on ΔT and MTD, respectively, as it was shown by Al Masri et al. [22].

Oerke et al. [49] reported the possibility to detect apple scab infection due to (*Venturia inaequalis*) before the symptoms become visible by IRT. The same was reported for downy mildew of cucumber and grapevine leaves [17,18]. In contrast, the early detection of other pathosystems was associated with higher temperatures. Gomez [50] reported higher temperatures of rose leaves infected with downy mildew (*Peronospora sparsa*) two days before symptoms become visible. The infection with *Tobacco mosaic virus* (TMV) caused an increase in leaf temperature of tobacco. This is due to the closure of stomatal cells because of the accumulation of salicylic acid after infection [51]. The higher sensitivity of IRT to detect leaf diseases pre-symptomatically compared with FHB could be attributed to the differences between the spikelet and the leaf structures. This results in differences between the transpiration systems and leads to a higher temperature of spikes compared with that of leaves [52]. IRT proved high potential not only in the early detection but it also provided a more accurate assessment of FHB severity than the visual assessment.

Biotic and abiotic stresses in plants have been widely studied using CFI during the last decade [53,54,55,56]. It provides a direct, non-invasive measurement of the photosynthetic apparatus and information about the impacts of fungal pathogens on host metabolism [55]. The most sensitive parameters, indicating downy mildew infection on grapevine (*Plasmopara viticola*) were Fv/Fm and Y [II] of PSII. It was possible to detect the infection 3 days before symptoms become visible [54]. Kuckenberg et al. [24] used Fm and Fv/F_0_ for early and precise detection of brown rust (*Puccinia recondita*) and powdery mildew (*Blumeria graminis*) on wheat. They reported that Fv/F_0_ was the most sensitive parameters responsive to both diseases. They were able to detect the infection before symptoms became visible or significant changes in the NDVI become pronounced. In the current study, Fv/Fm and Y [II] in addition to Fm showed also high sensitivity to monitor FHB. The potential of CFI to characterize FHB on wheat was investigated first by Bauriegel et al. [57]. They proved the possibility of Fv/Fm to discriminate between healthy and infected spikes first at BBCH 75. Using CFI, FHB was already detectable from 5 dai onwards which is in accordance with Bauriegel et al. [27,57]. However, the very early infection could be distinguished through a few pixels with lower Fm values (Figure 8). As already discussed, plugging of the rachis has a side effect on water movement upwards, detectable by IRT, but not by CFI. In contrast, CFI is sensitive to detect the local *Fusarium* infection at early stages (≤ 5%) when symptoms on glumes are difficult to be captured by IRT.

Hyperspectral imaging provides new insights in studying FHB compared with the other two sensors by investigating a broader spectral range from 400–2500 nm in narrow bands per pixel. This was pronounced in the spectral difference between infected spikelets and non-inoculated control along the electromagnetic spectrum. The high sensitivity of HSI has been proved for early detection of *Fusarium* infection before the symptoms become visible to the human eye [56]. Furthermore, HSI was used to quantify wheat resistance to FHB [15]. It was shown that the assessment of lesion phenotypes of *Cercospora* leaf spot by HSI can be a good reporter of sugar beet variety resistance [30,58]. Kuska et al. [29,59] investigated the resistance reaction of different barley genotypes to powdery mildew (*Blumeria graminis* f.sp. *hordei*). They proved the potential of HSI to characterize this pathosystem depending on resistance/susceptibility of the infected barley genotype.

The wavelength near 700 nm had the most pronounced response to *Cercospora* leaf spot of sugar beet. This response is due to the correlation between this wavelength and chlorophyll content [31]. In the current study, the effect of FHB infection was most pronounced for the wavelengths 500, 675, and 760 nm in the VIS-NIR range, and for wavelengths 1440, 1884, and 2000 nm in the SWIR range.

Spectral vegetation indices (SVIs) indicate specific parameters of plant functions. This reduces the data dimensionality and the computation time as well instead of considering the entire spectrum, [31,60]. However, a single vegetation index is not specific enough to differentiate between plant diseases or stress factors [31]. Huang et al. [28] correlated the DS to PRI and proved the potential of PRI to quantify yellow rust severity in winter wheat.

Out of the six vegetative indices used in this study, NDVI, PRI, PSSR (a, b and c), and WI [41,42,43,44], PSSRa and PSSRb had the highest sensitivity for early detection of *Fusarium* infection. This is in accordance with the study of Alisaac [15], which proved a high correlation between these indices and FHB infection on the spike scale. In the case of apple scab infection, Delalieux et al. [60] claimed that the performance of SVIs depends on disease development and leaf age. The presence of plant pigments including chlorophyll a and chlorophyll b plays a key role affecting spectral reflectance [61]. These pigments are controlled by the chemical and the biological activity of the host plant [62]. The second role affecting the spectral reflectance is the physical structure and the water content of the plant tissue [63]. Mahlein et al. [31] used PSSRa and PSSRb in combination with other SVIs to characterize *Cercospora beticola*, *Erysiphe beticola*, and *Uromyces betae* on sugar beet. They proved the possibility to differentiate between the three diseases using at least two indices in combination. As shown in this study, SVIs can be good indicators when they are correlated to each other and the parameters derived from CFI and IRT (Figure 7). This correlation can help to substitute one sensor by the other according to the application conditions in the field or the greenhouse.

An evaluation of the sensor’s feasibility to monitor plant diseases depends on the individual parameters of each sensor. Previous studies showed that HSI imaging technologies are more sensitive compared with non-imaging technologies [64]. A further factor influencing the data quality is the distance between the object and the sensor. It is therefore of high importance to identify a proper measuring setup for each individual sensor. In the current approach, optical sensors with different technical parameters were compared with each other based on their data.

A support vector machine (SVM) approach was applied by Alisaac et al. [15] to classify healthy and *Fusarium*-infected spikes using SVIs derived from HSI. They showed an increased accuracy starting from 79% at 4 dai to 95% at 17 dai. This is in accordance with the results of the current study that proved increasing accuracy from 78% at 3 dai to 100% at 12, 17, and 21 dai. The decrease in classification accuracy 30 dai is due to the senescence in the non-inoculated control spikelets.

In the current study, the highest classification accuracy of 89% was achieved based on the spectral vegetation indices of the spikelets derived from HSI. This was followed by parameters derived from IRT and CFI with classification accuracies of 82 and 79%, respectively. This confirms the results of Moshou et al. [65] who compared HSI with CFI to discriminate wheat leaves infected with yellow rust from healthy leaves. They showed that the classification accuracy using three bands from HSI was higher than using CFI parameters.

In the current approach, combining parameters from IRT and HSI gave the best improvement in the classification accuracy especially at 30 dai. Other combinations (i.e., IRT-CFI, CFI-HSI) gave no improvement if the classification accuracy compared with the individual sensor. This is in contrast with the results of Chaerle et al. [53] and Moshou et al. [65]. They proved that IRT provides a higher potential of non-invasive measurement when combined with CFI to characterize plant diseases. In addition, combining CFI with HSI parameters improved the accuracy of quadratic discriminant analysis (QDA) to 94.5% compared with the individual sensor when they were used to classify *Puccinia striiformis* infection. These results are due to the differences in the host plant and the pathogen in these studies.

Berdugo et al. [35] applied a discriminant analysis approach using multi-sensor data (IRT-CFI-HSI) on cucumber diseases. They proved the possibility of early discrimination of symptoms of *Cucumber mosaic virus* (CMV), *Cucumber green mottle mosaic virus* (CGMMV), and powdery mildew due to *Sphaerotheca fuliginea* on cucumber. In the current study, multi-sensor data of IRT-CFI-HSI did not improve the accuracy compared with the other combinations. This shows that the combination of IRT-HSI was superior not only to the other combinations but also to multi-sensor data (IRT-CFI-HSI) in monitoring FHB on wheat.

## 5. Conclusions

The present study showed that the use of different sensors allows to detect FHB infection on wheat spikelets and to monitor the damage of *Fusarium* species on wheat spikes. This can improve resistance phenotyping of wheat to FHB. Sensors data derived from IRT, CFI, and HSI showed a high correlation and combined, they are describing the development of disease severity. Data derived from HSI was most sensitive to identify the early response of wheat plants to FHB infestation followed by IRT and CFI. The combination of data derived from HSI-IRT gave superior accuracy over the time of the experiment, and it seems to be most promising. Sensor data can contribute substantially to the monitoring of FHB but the suitability of multi-sensor application under field conditions and on canopies of cereal will be the next step of investigations.

## Figures and Tables

**Table 1:** Summary of parameters derived from multi-sensorial monitoring of *Fusarium* head blight on wheat.

Sensor	Index	Equation	Indicator
Thermography (IRT)	Maximum temperature difference (MTD)	MTD = maximum − minimum temperature within spikelets	Biotic stresses in early stage [17]
	Average temperature difference (ΔT)	ΔT = average air temperature − average spikelets temperature	Biotic stresses in early and late stages [17]
Chlorophyll fluorescence imaging (CFI)	Maximal fluorescence yields	Fm	Fast chlorophyll fluorescence kinetics [24]
	Maximal PSII quantum yield (Fv/Fm)	Fv/Fm = (Fm − F_0_)/Fm	Maximal photochemical efficacy of photosynthesis II [39]
	Effective PSII quantum yield (Y [II])	Y [II] = (Fm’ − F)/Fm’	Photochemical quantum yields at steady state [40]
Hyperspectral imaging (HSI)	Normalized differences vegetation index (NDVI)	NDVI = (R_800_ − R_670_)/(R_800_ + R_670_)	Biomass, leaf area [41]
	Photochemical reflection index (PRI)	PRI = (R_531_ − R_570_)/(R_531_ + R_570_)	Epoxidation state xanthophyll’s cycle; pigments and photosynthetic radiation use efficiency [42]
	Pigment-specific simple ratio (PSSR)	PSSRa = R_800_/R_680_	Chlorophyll a [43]
		PSSRb = R_800_/R_635_	Chlorophyll b [43]
		PSSRc = R_800_/R_470_	Carotenoid [43]
	Water index (WI)	WI = R_900_/R_970_	Water content [44]

**Table 2:** Effect of *Fusarium* head blight caused by *F. graminearum* and *F. culmorum* on spectral vegetation indices of wheat spikelets compared with the non-inoculated control.

Index	Treatment	Time [dai]						
		3	5	7	12	17	21	30
**NDVI**	Non-inoculated control *F. graminearum* *F. culmorum*	0.77 a 0.72 a 0.75 a	0.76 a 0.70 a 0.75 a	0.77 a 0.68 a 0.72 a	0.77 a 0.52 b 0.55 b	0.76 a 0.47 b 0.46 b	0.74 a 0.38 b 0.40 b	0.55 a 0.30 b 0.24 b
**PRI**	Non-inoculated control *F. graminearum* *F. culmorum*	−0.01 a −0.02 a −0.02 a	−0.01 a −0.02 a −0.02 a	−0.02 a −0.03 b −0.03 b	−0.02 a −0.04 b −0.04 b	−0.02 a −0.05 b −0.05 b	−0.03 a −0.06 b −0.06 b	−0.06 a −0.06 a −0.06 a
**PSSRa**	Non-inoculated control *F. graminearum* *F. culmorum*	7.10 a 5.85 b 6.46 ab	6.72 a 5.29 b 6.56 a	7.21 a 4.96 b 5.81 b	6.99 a 3.29 b 3.48 b	6.63 a 2.81 b 2.73 b	6.29 a 2.28 b 2.39 b	3.50 a 1.86 b 1.57 b
**PSSRb**	Non-inoculated control *F. graminearum* *F. culmorum*	5.53 a 4.74 b 5.02 ab	5.27 a 4.42 b 5.11 a	5.58 a 4.24 b 4.65 b	5.44 a 3.03 b 3.16 b	5.19 a 2.67 b 2.59 b	4.94 a 2.23 b 2.33 b	2.99 a 1.90 b 1.64 b
**PSSRc**	Non-inoculated control *F. graminearum* *F. culmorum*	6.85 a 5.88 a 6.39 a	6.47 a 5.76 a 6.75 a	6.96 a 5.68 b 6.50 a	7.18 a 4.53 b 4.91 b	6.95 a 4.22 b 4.18 b	6.94 a 3.67 b 3.96 b	4.96 a 3.31 b 2.83 b
**WI**	Non-inoculated control *F. graminearum* *F. culmorum*	1.14 a 1.14 a 1.14 a	1.14 a 1.13 a 1.13 a	1.15 a 1.11 b 1.10 b	1.16 a 1.05 b 1.05 b	1.15 a 1.03 b 1.03 b	1.15 a 1.02 b 1.02 b	1.12 a 1.01 b 1.00 b

NDVI, normalized difference vegetation index; PRI, Photochemical reflection index; PSSRa, Pigment-specific simple ratio chlorophyll a; PSSRb, Pigment-specific simple ratio chlorophyll b; PSSRc, Pigment-specific simple ratio carotenoid; WI, Water index. Within one column, lowercase letters indicate significant differences between the parameters (Tukey’s HSD; P ≤ 0.05, n = 6).

**Table 3:** Accuracy of two-class classification (non-inoculated/infected spikelets) using support vector machine (SVM) for each assessment date using the defined parameters derived from each sensor and combinations of different sensors.

Time [dai]	Accuracy [%] of Two-Class Classification						
	IRT ^1^	CFI ^2^	HIS ^3^	IRT-CFI	IRT-HSI	CFI-HSI	Multi-Sensor (IRT-CFI-HSI)
**3**	78	56	78	67	67	56	56
**5**	100	67	78	67	100	78	100
**7**	78	89	89	78	78	78	78
**12**	78	89	100	100	100	100	100
**17**	100	89	100	100	100	100	100
**21**	78	89	100	89	100	100	100
**30**	67	78	78	67	89	89	78
**Mean**	82	79	89	81	90	86	87

^1^ IRT, infrared thermography; ^2^ CFI, chlorophyll fluorescence imaging; ^3^ HSI, hyperspectral imaging
